# The Transcription Factor *MafB* Regulates the Susceptibility of *Bactrocera dorsalis* to Abamectin via *GSTz2*

**DOI:** 10.3389/fphys.2019.01068

**Published:** 2019-08-20

**Authors:** Guang-Hui Tang, Ying Xiong, Yi Liu, Zhong-Hao Song, Yang Yang, Guang-Mao Shen, Jin-Jun Wang, Hong-Bo Jiang

**Affiliations:** ^1^Key Laboratory of Entomology and Pest Control Engineering, College of Plant Protection, Southwest University, Chongqing, China; ^2^State Cultivation Base of Crop Stress Biology for Southern Mountainous Land, Southwest University, Chongqing, China; ^3^International Joint Laboratory of China-Belgium on Sustainable Crop Pest Control, Academy of Agricultural Sciences, Southwest University, Chongqing, China

**Keywords:** oriental fruit fly, transcription factor, *MafB*, *GST*, abamectin

## Abstract

Pesticide resistance is a serious problem that poses a major challenge to pest control. One of the most potent resistance mechanisms is the overexpression of genes coding for detoxification enzymes. The expression of detoxification genes is regulated by a series of transcription factors. Previous studies have revealed that the increased expression of detoxification genes contributes to the insecticide tolerance of *Bactrocera dorsalis.* Our objective was thus to identify the transcription factors involved in this process. Temporal expression profiles showed that the transcription factor *MafB* and detoxification genes were expressed highly in the fat body. Further analysis showed that the expression of *MafB*, *GSTz2*, and *CYP473A3* was induced by abamectin. Disruption of the *MafB* transcription factor through RNA interference decreased the transcript levels of *GSTz2* and *CYP473A3* and increased the susceptibility to abamectin significantly. Direct silencing of the expression of *GSTz2* also increased susceptibility to abamectin, while *CYP473A3* did not. In conclusion, these results suggest that the expression of *GSTz2* and *CYP473A3* was regulated by the transcription factor *MafB*, and the up-regulation of *GSTz2* via *MafB* decreased the susceptibility of *B. dorsalis* to abamectin.

## Introduction

The oriental fruit fly *Bactrocera dorsalis* (Hendel) is globally distributed and constitutes one of the most damaging and economically important agricultural pests. In the field control of *B. dorsalis*, the application of insecticides has led to high levels of insecticide resistance in China as well as Hawaii of United States, which has resulted in severe ecological problems as well as significant economic losses (Hsu et al., 2004; Chou et al., 2010; Jin et al., 2014). Abamectin, a macrocyclic lactones compound, originated from the soil microorganism involving *Streptomyces avermitilis*, has been commonly used for the control of fruit flies (Jin et al., 2011). It belongs to the family of avermectins (Putter et al., 1981), targeting the glutamate-gated chloride channels (GluCls) and histamine-gated chloride channels (HisCls) (Zheng et al., 2002; McCavera et al., 2007). However, resistance selection analysis of a laboratory-reared strain indicated that *B. dorsalis* could develop high resistance to abamectin after 36 generations. Furthermore, resistance monitoring showed that field populations of *B. dorsalis* in China have developed resistance to abamectin (Jin et al., 2011). Therefore, investigating the underlying molecular mechanisms of insecticide resistance is an important prerequisite for the development of resistance management strategies.

Great progress has been made over the last two decades in understanding the molecular mechanisms of insecticide resistance. It has been well reported that decreased target site sensitivity, enhanced metabolic detoxification, and reduced cuticle penetration are the major contributors to resistance (Vontas et al., 2011; Liu, 2015; Vargas et al., 2015). Metabolic resistance refers to the enhanced detoxification and metabolism of insecticides in insects through increasing the expression or activity of detoxification enzymes. Notably, the overexpression of cytochrome P450 monooxygenases (P450s), carboxylesterases (CarEs), and glutathione *S*-transferases (GSTs) constitutes the most common metabolic resistance mechanism (Wilson, 2001). RNA interference (RNAi) technology can effectively detect the importance of these continuously overexpressed detoxification genes in insect resistance. Interference of the overexpressed genes (*CYP6FD2* and *CYP6FF1*) of *Locusta migratoria* has led to an increase in deltamethrin toxicity (Guo et al., 2016). In *Tetranychus cinnabarinus*, P450 genes, such as *CYP389B1* and *CYP392A26*, were overexpressed in a fenpropathrin-resistant strain (Shi et al., 2015). The transcription levels of *GSTs* (*GSTe2*, *GSTe4*, and *GSTe9*) and *CarEs* (*CarE2*, *CarE4*, and *CarE6*) in malathion-resistant strains of *B. dorsalis* were increased, and the silencing of these genes by RNAi could increase the susceptibility to malathion (Wang et al., 2015, 2016; Lu et al., 2016).

To further clarify the regulatory mechanisms of specific genes, the transcription system of xenobiotic detoxification in *Drosophila* was assessed (Misra et al., 2011). Under stress exposure conditions, Keap1 releases CncC [a basic leucine zipper (bZIP) protein], which translocates to the nucleus, and a heterodimer formed by CncC and sMaf (belonging to the bZIP family) binds upstream of the stress-response genes (XRE) to regulate the expression of antioxidant genes (Nioi et al., 2003; Baird and Dinkova-Kostova, 2011; Misra et al., 2011; Hirotsu et al., 2012). It has been reported that the Cncc–Maf heterodimer is involved in the transcriptional regulation of detoxifying metabolic enzymes. In *Drosophila melanogaster*, *CYP6a2* and *CYP6a8* were down-regulated following the knockdown of CncC, which resulted in a decreased DDT tolerance in DDT-resistant strains (Misra et al., 2013). Expression of *GSTd1* in *D. melanogaster* was also reduced after the silencing of CncC and led to the high mortality of paraquat poisoning (Sykiotis and Bohmann, 2008). In *Aphis gossypii*, CncC regulates *Cyp6AD2* to alter gossypol tolerance in a gossypol-resistant strain (Peng et al., 2016).

Oriental fruit flies are polyphagous pests that reduce the yield and quality of fruit, often cause spoilage. The long-term intensive use of pesticides has led to resistance to various insecticides in the species (Brown and Payne, 1988). It was previously indicated that the overexpression of detoxification enzymes constitutes a primary mechanism of resistance in *B. dorsalis.* In this study, we focused on the function of the transcription factor *MafB* and its downstream genes, which are involved in the abamectin tolerance of *B. dorsalis*.

## Materials and Methods

### Test Insects

Larvae of the flies were originally collected from the infested oranges in Hainan province, China, in 2008. For this study, 60 generations of the flies have been reared. The insects were reared with an artificial diet according to a previously described protocol (Wang et al., 2013). The laboratory strain of the flies was cultured in a growth chamber at 27.5 ± 0.5°C, 75 ± 5% relative humidity, and a photoperiod of 14:10 h (light:dark).

### Molecular Cloning

The reference sequence of the *MafB* cDNA was acquired from NCBI (GenBank accession number, XM_011207424). Primers amplifying the complete open-reading frame (ORF) were designed (Table 1) using Primer Premier 5.0 (Premier Biosoft International, Palo Alto, CA, United States). PrimeSTAR high-fidelity DNA polymerase (Takara, Dalian, China) was used for PCR amplification. The purified PCR product was subcloned into the pGEM-T Easy Vector (Promega, Beijing, China), and the construct was transformed into Trans5α chemically competent cells. Positive clones were sent for sequencing (TransGen Biotech, Beijing, China).

**TABLE 1:** Primers for *MafB* used in the study.

**Experiment**	**Primer names**	**Sequences (5′–3′)**
Full-length	*MafB*-F	ATGAGAATGGAAGACCCAAACC
	*MafB*-R	GCAAGTTGGCGTCAGAGA
RT-qPCR	*MafB*-qF	GAAATCTACACGCACGGACC
	*MafB*-qR	AGGATCTGAACGCATGGAGT
dsRNA synthesis	*MafB*-dsF	TAATACGACTCACTATAGGG AATGGAAG TACTTGTGCCCAAA
	*MafB*-dsR	TAATACGACTCACTATAGGG GCTATATTG TCTGGAGCAGGT

**MafB*, transcription factor. The underlined letters represent the T7 promoter sequences for efficient *in vitro* transcription in dsRNA synthesis.*

#### Quantitative Reverse Transcript PCR

RNA was extracted from different tissues, including the Malpighian tubules, fat body, and midgut. TRIzol reagent (Life Technologies, Carlsbad, CA, United States) was used for the RNA isolation with a genome DNA elimination by DNase I (Promega, Madison, WI, United States). The extracted RNA was purified using phenol/chloroform method and dissolved in the RNase-free water. The concentration and the purity were assessed by Nanodrop 1000 (GE Healthcare Bio-sciences, Uppsala, Sweden). The cDNA synthesis was conducted by the PrimeScript 1st Strand cDNA Synthesis Kit (Takara, Dalian, China) according to the manufacturers’ instructions. For each sample, 1 μg of total RNA was used to synthesize cDNA. A serial dilution (fivefold) of the cDNA was employed to determine the amplification efficiency of each primer pair for the target gene. Quantitative Reverse Transcript PCR (RT-qPCR) was performed in 10 μL reaction volumes consisting of 5 μL qPCR Master Mix (Promega, Madison, WI, United States), 0.5 μL each of cDNA and reverse primers (5 μM), and 3.5 μL nuclease-free water. The RT-qPCR reactions were conducted with the following program: initial incubation of 95°C for 2 min, followed by 40 cycles of 95°C for 15 s and 60°C for 30 s. RPS and α-tubulin served as the internal reference genes for normalization of the relative expression levels (Shen et al., 2010). The RT-qPCR was conducted with two technical and four biological replications. The relative expression data were analyzed using Qbase. The expression of *MafB* and detoxification enzymes in three different tissues of *B. dorsalis* was log2 transformed. A heat map was generated by HemI. RNAi and abamectin treatment data are presented as the mean ± standard error (SE). All the data were subject to independent *t-*test by utilizing SPSS 20.0 (SPSS Inc., Chicago, IL, United States).

### Phylogenetic Analysis and Identification

MafB protein sequences of dipteran species were obtained from the NCBI web server^1^ and aligned with the MafB sequences generated in the present study using Clustal (Larkin et al., 2007) and JalView 2.9 (Waterhouse et al., 2009). To infer the evolutionary relationships, the neighbor-joining method was used to construct a phylogenetic tree in MEGA5.05 (Tamura et al., 2011) with 1000 bootstrap replicates. The molecular weight and isoelectric point of MafB were predicted using the online software tool^2^.

### RNAi

A fragment of *MafB* was amplified by PCR using primers (Table 1) containing the T7 promoter as the template for double-stranded (ds) RNA synthesis. The dsRNA was synthesized using the TranscriptAid T7 High Yield Transcription Kit (Thermo Scientific, Wilmington, DE, United States). About 1.5 μg of dsRNA was injected into the abdomen between the first and second abdominal segments with a Nanoject II Auto-Nanoliter Injector (Drummond Scientific, Broomall, PA, United States). Equivalent injection quantities of the ds green fluorescent protein (dsGFP) served as the control. The RNAi efficiency was determined using randomly collected adults at 24 and 48 h post-injection. At least 60 4-day-old adult flies were used for each treatment and control group. Mortality was less than or equal to 5% after dsRNA injection for each treated and control group.

### Abamectin Bioassay

Abamectin (95% purity) was purchased from Bangnong Chemical Company (Guangzhou, China). The bioassay was performed following the knockdown of *MafB*, *CYP437A3*, and *GSTz2*. A 0.5-μL of abamectin [concentration = LC_40_ (105 μg/mL)] was applied to the pronotum of test insects using a PB-600-1 repeating dispenser (Hamilton Company, Reno, NV, United States). For the dsRNA-injected flies, the bioassay was conducted at 24 h after the injection. At least 60 injected flies were used for the bioassays for each treatment. Mortality of the flies was scored after 24 h of exposure to abamectin. Chi-square (χ^2^)-test was used to determine the significant difference of the mortality between dsRNA treated and control group by utilizing SPSS 20.0 (SPSS Inc., Chicago, IL, United States). Data are presented as the mean ± SE.

## Results

### *MafB* Sequence Analysis

**FIGURE 1:**
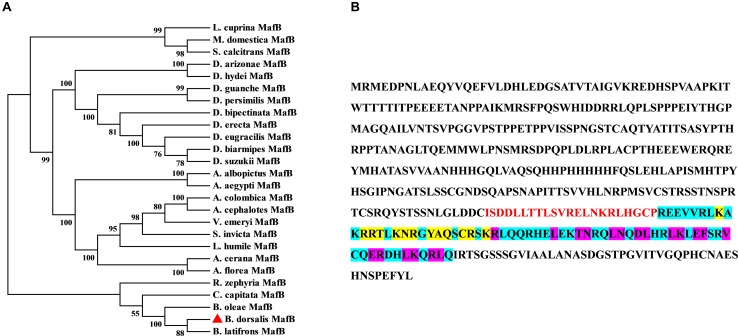
Sequence and phylogenetic tree of *MafB*. **(A)** Phylogenetic tree of MafBs. Amino acid sequences of *B. dorsalis MafB* and dipteran *MafB* were aligned to forming phylogenetic tree to assess their relationships. *MafB* of *B. dorsalis* was indicated by the red rectangle. **(B)** The amino acid sequence structure of *MafB.* The basic leucine and zipper (bZIP) domain are indicated by blue background and red color. DNA-binding site and dimer interface domain are indicated by yellow and purple core color, respectively.

The ORF of *MafB* was screened out from the transcriptome data of *B. dorsalis* and confirmed by PCR. The sequence of *MafB* is accessible in GenBank (XM_011207424). The complete ORF of *MafB* contains 1323 nucleotides and encodes 440 amino acids. The predicted molecular weight of the MafB protein is 48.71 kD, and the isoelectric point is 6.97. The phylogenetic tree of dipteran *MafB* protein sequences shows that the *MafB* proteins are relatively conserved and that the *MafB* of *B. dorsalis* is close to the *MafB* of *B. latifrons* and *B. oleae* from Tephritidae (Figure 1). The *MafB* protein includes one conserved domain (Basic Leucine zipper domain). There is a dimerization domain and a DNA-binding site (DBD) (Figure 1). Thirteen residues consist of DNA-binding sites and 19 are composed of a dimer interface that can form a dimer and bind upstream of the target gene to initiate transcription.

### Tissue-Specific Expression Profiling

**FIGURE 2:**
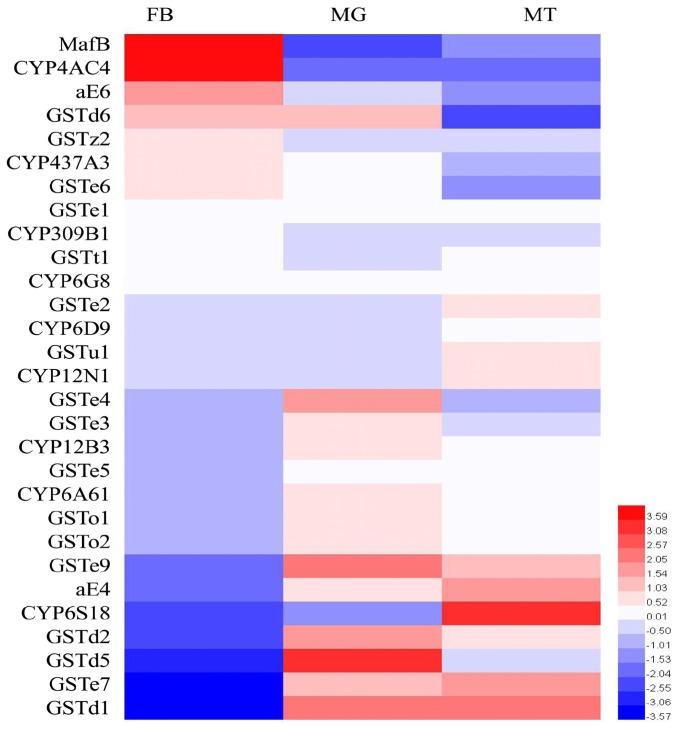
The transcriptional expression profiles of *MafB* and detoxification enzymes in three different tissues of *B. dorsalis*. MT, Malpighian tubules; MG, midgut; FB, fat body. The expression of *MafB* and detoxification enzymes of *B. dorsalis* was log2 transformed. Different colors represent the degree of expression, red and blue indicate upregulation and downregulation, respectively.

The expression patterns of the transcription factor (*MafB*) as well as the detoxification enzymes in the three major tissues (midgut, fat body, and Malpighian tubules) associated with insecticide metabolism were analyzed by RT-qPCR (Figure 2). The highest expression of *MafB* was detected in the fat body, followed by the midgut and then the Malpighian tubules. Based on this characteristic, *GSTe6*, *GSTz2*, *CYP437A3*, *CYP4AC4*, and *aE6*, which were highly expressed in the fat body, were screened out. These filtered genes were then used in the following experiments.

### Abamectin-Induced Expression

**FIGURE 3:**
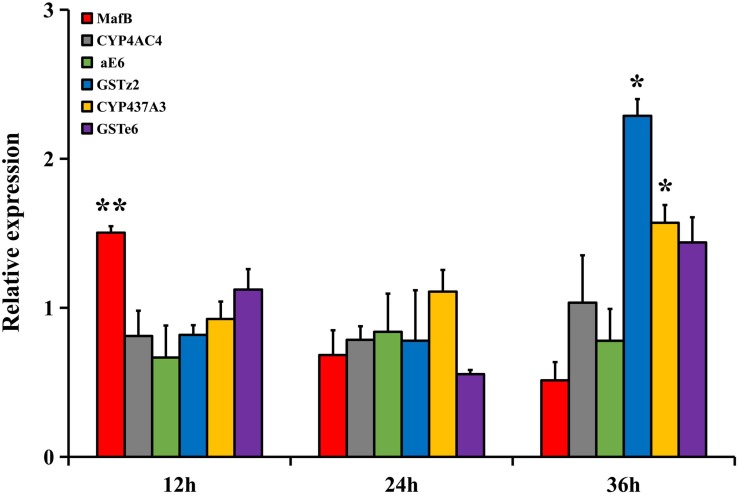
The transcriptional expression profiles of *MafB* and *GSTe6*, *GSTz2*, *CYP4AC4*, *CYP437A3*, and *aE6* of *B. dorsalis* in the fat body by abamectin treatment. The data shown are mean ± SE (*n* = 3). Relative expression was calculated based on the value of acetone. Asterisks (^∗^ and ^∗∗^) above the error bars present statistical differences determined by the independent samples *t*-test with *P* < 0.05 and *P* < 0.01, respectively.

The relative expressions of *MafB* and the filtered detoxifying genes in the 4-day-old adult flies under abamectin treatment at three different time points (12, 24, and 36 h) were analyzed by RT-qPCR (Figure 3). The relative expression of *MafB* in the fat body after treatment was significantly higher than in the control at 12 h (*P* < 0.05), indicating the rapid response of *MafB* to abamectin stimulation. The relative expressions of *CYP437A3* and *GSTz2* in the fat body after 36 h of treatment were significantly increased (*P* < 0.05). However, the expression of *CYP4AC4*, *aE6*, and *GSTe6* did not change significantly at any time point following abamectin treatment. *CYP437A3* and *GSTz2* were thus selected for the subsequent interference experiments.

### RNAi of *MafB* and Abamectin Susceptibility Test

**FIGURE 4:**
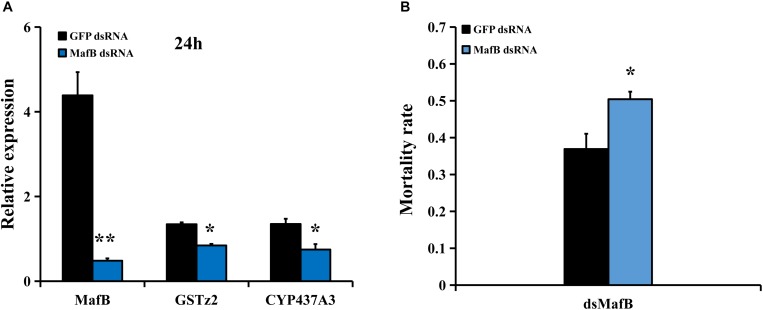
Susceptibility of *B. dorsalis* to abamectin after *MafB* knockdown by RNAi. **(A)** Relative expression levels of *MafB* after *B. dorsalis* adults were injected with ds*MafB* compared to ds*GFP*, a double-stranded green fluorescent protein RNA. **(B)** Mortality rate for the ds*MafB*- and ds*GFP*-injected flies after abamectin treatment. Asterisks (^∗^ and ^∗∗^) above the error bars present statistical differences determined by the independent samples *t*-test with *P* < 0.05 and *P* < 0.01, respectively.

To examine whether *MafB* controls the expression of detoxifying genes (including *CYP437A3* and *GSTz2*) and affects the susceptibility of abamectin, RNAi of *MafB* was conducted. The suppression efficiency of *MafB* compared to the *GFP*-injected control was quantified by RT-qPCR (Figure 4A). The transcriptional level of *MafB* was significantly reduced (88.4% suppressed) than in the control after 24 h (*P* < 0.05). RNAi of *MafB* also led to the reduced expression of two detoxification genes (*CYP437A3* and *GSTz2*). The abamectin bioassays showed that RNAi of *MafB* presented a significantly higher mortality rate (Figure 4B). Under abamectin exposure, the mortalities at 24 h were 37 and 51% in the flies injected with dsGFP and ds*MafB*, respectively.

### RNAi of *GSTz2* and *CYP437A3* and Abamectin Susceptibility Test

**FIGURE 5:**
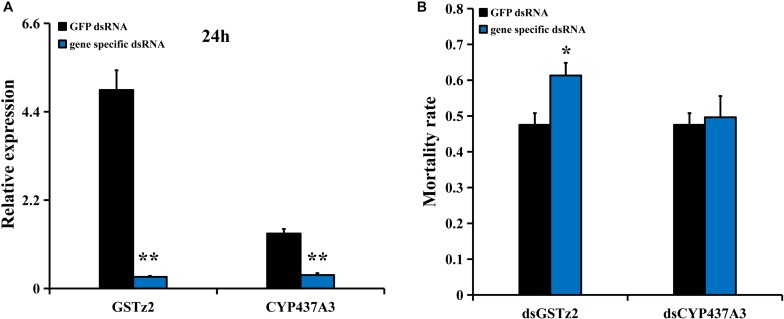
Susceptibility of *B. dorsalis* to abamectin after *GSTz2* and *CYP437A3* knockdown by RNAi. **(A)** Relative expression levels of *GSTz2* and *CYP437A3* after *B. dorsalis* adults were injected with ds*GSTz2* and ds*CYP437A3*, respectively, compared to ds*GFP*, a double-stranded green fluorescent protein RNA. **(B)** Mortality rate for the ds*GSTz2* and ds*CYP437A3* and ds*GFP*-injected flies after abamectin treatment. Asterisks (^∗^ and ^∗∗^) above the error bars present statistical differences determined by the independent samples *t*-test with *P* < 0.05 and *P* < 0.01, respectively.

RNA interference of *CYP437A3* and *GSTz2* was conducted in *B. dorsalis* using the same method described above. The mRNA levels of *CYP437A3* and *GSTz2* were significantly reduced after 24 h (*P* < 0.05) (Figure 5A). The abamectin bioassay suggested that the RNAi of *GSTz2* was associated with a higher mortality rate (Figure 5B). However, the RNAi of *CYP437A3* had no effect on mortality. Under exposure to abamectin, the mortalities at 24 h were 47 and 61% in the flies injected with ds*GFP* and ds*GSTz2*, respectively.

## Discussion

The principal objective of this study was to confirm whether *MafB* functions in the regulation of detoxification genes in *B. dorsalis*. Although small Maf works as a ligand protein of CncC, it plays an indispensable role in the heterodimer that regulates the detoxification of metabolic enzymes. Without Maf, the regulatory pathway of CncC is invalid. When CncC and Maf were both expressed in *Tribolium castaneum*, luciferase activity was higher than under the expression of CncC alone in the regulation of *CYP6BQ12*, *CYP6BQ6*, *CYP6BQ7*, and *CYP6BQ9* (Kalsi and Palli, 2015). In *T. cinnabarinus*, luciferase activity was also higher in the presence of both CncC and Maf than in the presence of CncC alone in the regulation of *CYP389B1* and *CYP392A28* (Shi et al., 2017). In addition, the bZIP nuclear transcription factor family is composed of small Maf (MafG, MafK, and MafF) and large Maf (c-Maf and MafB) proteins that repress as well as activate the transcription of many genes. Notably, large Maf (MafB) and CncC proteins belong to the bZIP family. Large Maf (MafB) proteins are similar to CncC proteins and possess a transcription activity region, binding to antioxidant response elements (AREs) to activate or repress ARE-mediated transcription (Katsuoka and Yamamoto, 2016). It has been documented that large Maf proteins regulate the gene expression of ARE-mediated detoxifying enzymes (Saravanakumar and Jaiswal, 2002). The functional analysis of *MafB* is thus particularly important in oriental fruit fly.

In order to explore the functions of *MafB*, the expression of *MafB* was analyzed. Compared with the midgut and Malpighian tubules, the *MafB* gene was highly expressed in the fat body and exhibited tissue specificity. The fat body is one of the most important tissues for detoxification metabolism. Numerous P450s express in the fat body, midgut, and Malpighian tubules. Signaling of the *CYP4p2* and *CYP4s3* genes has been exclusively detected in the fat body via a hybridization technique (Chung et al., 2009). The fat body plays a significant role in metabolic functioning, including in the storage and delivery of energy to satisfy energy demands in insects (Arrese and Soulages, 2010). The high expression in the fat body implies that *MafB* was involved in detoxification metabolism. Based on the transcripts, *CYP4AC4*, *CYP437A3*, *aE6*, *GSTe6*, and *GSTz2* were highly expressed in the fat body, which suggests a regulatory relationship between *MafB* and these genes.

Abamectin treatment experiments were then conducted to further explore these regulatory relationships. *MafB* was significantly expressed at 12 h in the fat body following abamectin treatment, and the downstream target genes (*GSTz2* and *CYP437A3*) were elevated at 36 h, which indicates that *MafB* responds to abamectin more rapidly and that *MafB* is likely to regulate downstream genes. However, the mRNA levels of *GSTe6*, *aE6*, and *CYP4AC4* were not significantly increased at any time following abamectin treatment. RNAi experiments were performed to further evaluate the relationship between *MafB* and downstream genes (*GSTz2* and *CYP437A3*).

RNA interference was used to disrupt the expression of *MafB*. *MafB* knockdown resulted in increased abamectin susceptibility and the down-regulation of *CYP437A3* and *GSTz2*. In a previous experiment, the transcription levels of detoxification genes were suppressed by Maf disruption, including *CYP6M2*, *GSTD1*, *GSTD3*, *jheh1*, *jheh2*, and *Gnmt*, leading to increased susceptibility to DDT, permethrin, and deltamethrin in *Anopheles gambiae* (Ingham et al., 2017). Furthermore, Maf-S transcription factors were found to control the expression of P450 genes associated with deltamethrin resistance in *T. castaneum* (Kalsi and Palli, 2015), imidacloprid resistance in *Leptinotarsa decemlineata* (Kalsi and Palli, 2017), and fenpropathrin resistance in *T. cinnabarinus* (Shi et al., 2017). The studies indicated that the silencing of Maf results in the down-regulated expression of detoxifying enzyme genes, which further influences metabolism. In this paper, we found that the mRNA levels of *GSTz2* and *CYP437A3* were also significantly decreased following *MafB* knockdown. We also demonstrated that the silencing of *GSTz2* results in increased susceptibility to abamectin. GSTs are an important group of detoxification enzymes and can be divided into six classes, including epsilon, delta, zeta, theta, omega, and sigma (Ding et al., 2003). *GST* activity was increased in the fat body and hemolymph under exposure to abamectin in Colorado potato beetle (Tomilova et al., 2016). In pollen beetle *Meligethes aeneus*, cytochromes P450 and GST made a contribution to deltamethrin exposure (Erban et al., 2017). However, silencing of *CYP437A3* did not alter the susceptibility of *B. dorsalis* to abamectin in the current study, indicating that it is a downstream gene of *MafB* and may not contribute decisively to abamectin susceptibility.

## Conclusion

In conclusion, the results indicated that the expression of *GSTz2* was regulated by the transcription factor *MafB*, which resulted in the susceptibility change of *B. dorsalis* to abamectin. This study improves our understanding of the regulatory relationship between *MafB* and detoxification enzymes and also confirms that *GSTz2* is an important downstream gene that is involved in abamectin tolerance.

## Data Availability

All datasets for this study are included in the manuscript and/or the supplementary files.

## Author Contributions

H-BJ and J-JW designed the research and provided the materials and reagents. G-HT, YX, YL, and YY performed all of the experiments. Z-HS and G-HT reared the test insects and collected the data in bioassay. G-HT, YX, G-MS, and H-BJ analyzed the data. G-HT wrote the original draft. H-BJ, J-JW and G-MS edited and modified the manuscript.

## Conflict of Interest Statement

The authors declare that the research was conducted in the absence of any commercial or financial relationships that could be construed as a potential conflict of interest.

## Footnotes

1 http://www.ncbi.nlm.nih.gov

2 http://web.expasy.org/protparam/
